# Development of Chincho (*Tagetes elliptica* Sm.) Essential Oil Organogel Nanoparticles through Ionic Gelation and Process Optimization with Box–Behnken Design

**DOI:** 10.3390/gels8120815

**Published:** 2022-12-11

**Authors:** Francis Cerrón-Mercado, Bettit K. Salva-Ruíz, Diana Nolazco-Cama, Clara Espinoza-Silva, Juana Fernández-López, Jose A. Pérez-Alvarez, Manuel Viuda-Martos

**Affiliations:** 1 IPOA Research Group, Agro-Food Technology Department, Centro de Investigación e Innovación Agroalimentaria y Agroambiental (CIAGRO-UMH), Miguel Hernández University, Orihuela, 03312 Alicante, Spain; 2 Departamento de Tecnología de Alimentos y Productos Agropecuarios (TAPA), Universidad Nacional Agraria la Molina, UNALM, Lima 15024, Peru; 3 Centro de Investigación de Productos Naturales, Universidad Nacional del Centro del Perú, UNCP, Lima 15024, Peru

**Keywords:** essential oil, *Tagetes elliptica* Sm., Box–Behnken design, chitosan nanoparticles, antibacterial activity

## Abstract

The aim of this work was to obtain chitosan nanoparticles (<1000 nm) with chincho (*Tagetes elliptica* Sp.) essential oil (CEO-CSNPs) using the ionic gelation method. A Box–Behnken design (BBD) was applied, using chitosan solution (CS) pH (4.0, 4.4, 4.8); the mass ratio of CS/CEO (1:0.7, 1:0.85, 1:1.0) and the mass ratio of CS/CS-tripolyphosphate (1:0.46, 1:0.58, 1:0.7) as independent variables. The formulation-dependent variables, encapsulation efficiency (EE) and loading capacity (LC) of the CEO-CSNPs were evaluated. BBD determined that optimal conditions for CEO-CSNPs were pH: 4.4, CS/CEO mass ratio 1:0.7 and CS/TPP mass ratio 1:0.46. Once the optimization was defined, particle size (PS), zeta potential (ZP), polydispersity index (PDI), CEO-CSNPs morphological studies, in vitro CEO release, and antibacterial activity were determined. The CEO-CSNPs showed an EE of 52.64% and a LC of 11.56%, with a diameter of 458.5 nm, with a ZP of 23.30mV, and a PDI of 0.418. The SEM studies showed that the nanoparticles were rounded and had uniform shapes. In addition, CEO-CSNPs showed a minimum inhibitory concentration against *Staphylococcus aureus, Salmonella infantis* and *Escherichia coli* of 5.29, 10.57 and 10.57 µg/mL, respectively. These results could be very useful for the stabilization of chincho essential oil for food industry purposes. However, several studies about the release, as well as interaction with food matrices, will be necessary.

## 1. Introduction

At present, consumers demand healthy foods in which artificial additives, preservatives, and colorants, among other additives, tend to be reduced or eliminated. Thus, the use of spices and derived products such as essential oils has been increased by the industries [1,2]. The species *Tagetes elliptica* Sm. known as “chincho”, is one of the most consumed herbs in Peru. This herb is used for its culinary quality (gourmet) and is incorporated in stews and dressings with meat, among other foods. Nevertheless, this spice is locally consumed, and it is not well known to the research community and consumers [3,4,5]. Essential oils (EO) are hydrophobic, volatile liquids extracted from plants, with a high content of bioactive compounds, including terpenes, terpenoids, phenol derivatives, and aliphatic compounds [6,7]; they have been used in the food industry as preservatives due to their antioxidant and antimicrobial activity [8,9,10], in addition to their antimicrobial activity effect [7].

There is evidence that other well-known plants of the genus *Tagetes*, including *T. minuta*, contain bioactive compounds with antioxidant properties [11,12]. *T. multiflora* has several volatile substances, such as terpenes among the main ones, to β-ocimene, dihydrotagetenone, (Z) tagetone, and limolene [11,13,14] that contribute to its special flavor and odour characteristicas. *T. erecta* has been used as a coloring agent and nutritional supplement [15] and is also used as a natural antioxidant in foods [16]. Due to its essential oils, *T. elliptica* Sm. could be used as natural additive for the food industry [17,18,19]. Although not many studies on this spice (*T. elliptica*) are reported so far, it has been evidenced that chincho essential oil (CEO) has antibacterial activity against *Staphylococcus aureus*, *Staphylococcus epidermidis* [20,21], *Bacillus subtilis*, *Escherichia coli*, and *Pseudomonas aeruginosa* [21]. The major compounds found in *Tagetes elliptica* essential oil are β-myrcene, cis/trans—epoxymyrcene and trans-tagetenone [20,22,23]. All of these compounds give a strong flavor and taste that make, for the Peruvians, a gourmet spice. CEO could be a natural alternative to synthetic additives, however, its use is limited, as CEO is susceptible to degradation due to its physical characteristics (color degradation, lipid oxidation, among others), compound volatility, and chemical degradation [24].

Encapsulation technology has been investigated to improve the physical stability, solubility, retention of bioactive properties, sensory properties, and shelf life of essential oils [25,26]. An important aspect that must be considered is the selection of the polymeric matrix. Thus, the matrix must be suitable to form the nanoparticles. In this sense, chitosan (CS), as a food polysaccharide, is non-toxic, biocompatible, and biodegradable with good barrier properties, and is the second most abundant biopolymer in nature [27]. It has been used as an encapsulant with emulsion-forming properties that can generate core-wrap constituents [28]. In addition, it is interesting for its antibacterial activity against several bacterial strains, including *Staphylococcus aureus* and *Escherichia coli* [29]. Due to all these characteristics, chitosan is a promising candidate to structure oleogels. Nevertheless, it is important to highlight that chitosan showed a low oil solubility. This fact should be compensated for to allow the formation of an adequate tridimensional network to retain the oil, thus forming a gel as mentioned Brito et al. [30].

Since essential oils are lipophilic and are a great challenge for food industry applications, the colloidal dispersion elaboration could have interesting advantages for essential oil availability in a food aqueous matrix, by avoiding their incompatibility in these food systems. Organogels are semi-solid systems in which a tridimensional network is composed of cross-linked gelators and fibres immobilized in an organic liquid (essential oil). According to Carrancá Palomono et al. [31] and Esposito et al. [32], the dispersions of organogel emulsions from hydrocolloids have promising stability properties, are easily formulated, and can be produced with natural organogelators, as chitosan, and could give them a great application for the food industry. Organogel and nanoparticle technology are systems that look for the increase in bioavailability for lipophilic substances, such as essential oils, in aqueous systems [33,34]. Encapsulation by ionic gelation is a suitable method to encapsulate essential oils with different types of active principles [35,36]. It has also proven its efficiency to maintain the thermal stability of the phenolic content in peppermint and green tea essential oils [29]. This technique increases the antioxidant and antimicrobial activity, even with an encapsulation efficiency rate of 40.6% [37]. Due to their nanometric size, these nanoparticles (NPs) in the use of ionic gelation microencapsulation can enhance their bioefficiency (bioavailability and solubility in aqueous media). However, the influence of the dependent and independent variables/factors involved in or during the process could affect the physicochemical properties of nanoparticle formation [38]. To optimize all factors and variables that can affect ionic gelation microencapsulation, the applied experimental designs can be programmed utilizing the Response Surface Methodology (RSM) and the Box–Behnken Design (BBD). RSM is an excellent tool that is very useful for detecting the influence of different formulation factors on response variables [39,40], while BBD is applied to establish the optimal experimental conditions, with optimal polynomial equations for the evaluated response variables [41,42].

The aim of this study was the development of a chincho essential oil nanoparticle (CEO-CSNP) organogel system through the application of RSM and BBD strategies, and to optimize the encapsulation efficiency (EE) and loading capacity (LC) of chincho essential oil nanoparticles (CEO) using, as a matrix, chitosan and chitosan (CS) sodium tripolyphosphate (CS-TPP). In addition, it was to study the influence of CS-pH and the mass ratio between CS-CEO mass and CS-TPP, and to analyze the hydrodynamic particle size, zeta potential, PDI, and particle morphology using Scanning Electron Microscopy (SEM). The efficiency of these experiments was evaluated by in vitro release studies and antimicrobial activity. 

## 2. Results and Discussion

### 2.1. Experimental Design Summary

#### 2.1.1. Fitting Model

Box–Benhken design was applied to optimize CEO-CSNPs. A total of 17 runs with 5 center points were performed to assess the effect of 3 independent variables, which can be observed (Table 1). As independent variables, we considered the pH of chitosan solutions (X_1_); Chitosan mass ratio (*w*/*v*) between chitosan-CH and chincho essential oil-CEO (X_2_); and mass ratio (*w*/*w*) between chitosan-CS and sodium Tripolyphosphate-TTP (X_3_), over two dependent variables: encapsulation efficiency (EE) (%) obtained from equation 2 as Y_1_, and loading capacity LC (%) calculated using Equation (3) as Y_2_ This experimental design can be observed in Table 1. All parameters under study and their respective values of this BBD can be observed in material and methods section.

#### 2.1.2. Statistical Analysis

For all the dependent variables (encapsulation efficiency—Y_1_, and loading capacity—Y_2_), the analysis of variance was carried out using the Box–Behnken design (BBD), and certain parameters, including *p*-value, F-value, and model F-value, were obtained using ANOVA applying the Design Expert software. The best model to fit for all response variables was the quadratic one, if compared to all other models (first-order), and was validated using the ANOVA (multifactor). The ANOVA summary for the quadratic model is presented in Table 2. It was noticed that the *p*-value for all responses (Y_1_ and Y_2_) was <0.0001, which is necessary for confirming that the independent variables showed a significant effect on the investigated response variables. The F-value of the response variables, it was shown that higher values are recommended to provide few errors in the model. The model F-values for Y_1_ and Y_2_ were 58.10 and 106.09, respectively, which shows a significant model. Due to residual noise, there is only a 0.01 percent chance that this large model F-value will occur. Concerning the lack of fit, it is mandatory to be non-significant in order to fit the model and confirm its efficiency [43].

### 2.2. Effect of Independent Variables on Encapsulation Efficiency (Y_1_)

The encapsulation efficiency is a very important parameter, since essential oils are chemically and physically unstable during their shelf-life, which could limit their use for new food formulations [24]. These technical problems could be solved using encapsulation techniques. Thus, the essential oils can act as antimicrobials and antioxidants in food processing, improving food safety and shelf-life [37,44].

The encapsulation efficiency (%) of various formulation chincho essential oil nanoparticles organogel system was determined, and values obtained were summarized in Table 1. The formulation EE% ranged between 12.15 ± 0.44 and 52.92 ± 0.65. It very important to notice that a reverse relation was found between the encapsulation efficiency of chincho essential oil nanoparticles organogel system formulations and the concentration of the different independent variables used. A positive or negative value is related to a positive or negative effect on the studied response (Y_1_), respectively.

Encapsulation Efficiency (Y_1_):

20.78 − 5.79 X_1_ − 1.51 X_2_ − 8.16 X_3_ − 2.40 X_1_X_2_ + 6.28 X_1_X_3_ + 4.77 X_2_X_3_ − 2.74 X_1_^2^ + 3.09 X_2_^2^ + 12.56 X_3_^2^ (1)

**Figure 1:**
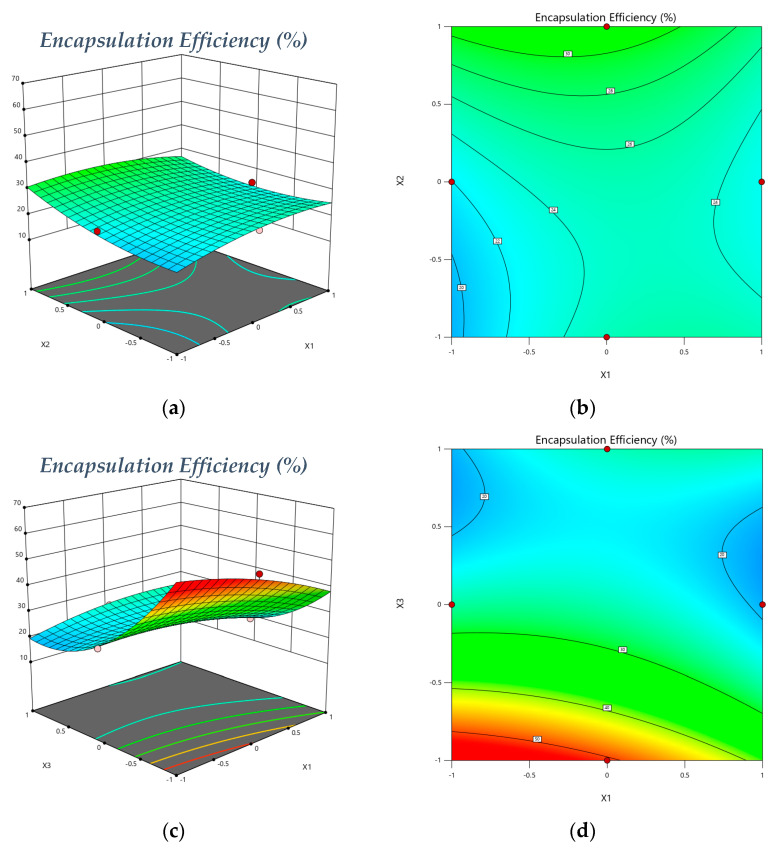

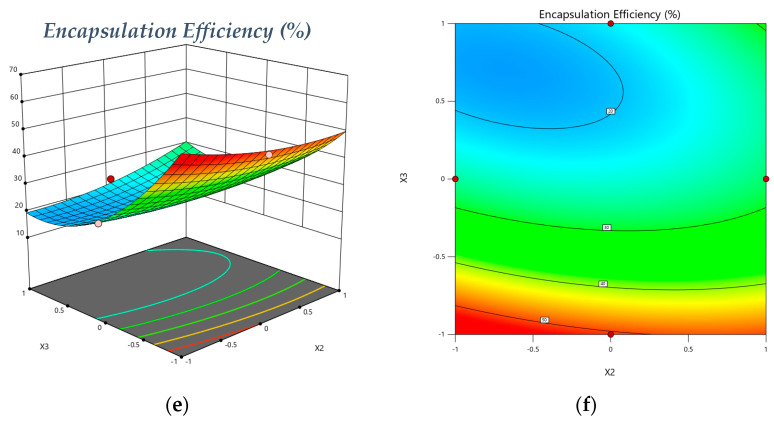
Model graphs: Encapsulation Efficiency. (**a**) Three-dimensional response surface plots showing the effect of variables (X_1_ = pH (4.4) and X_2_ = Chitosan: Chincho essential Oil (*w*/*v*) (1: 0.7)) on response (Encapsulation Efficiency), (**b**) 2D contour graph, (**c**) 3D response surface plots showing the effect of variables (X_1_ = pH (4.4) and X_3_ = Chitosan: TPP (*w*/*w*) (1:0.46)) on the response variable (Encapsulation Efficiency, (**d**) 2D contour graph, (**e**) 3D response surface plots showing the effect of variables (X_2_ = Chitosan: TPP (*w*/*w*) (1:0.7) and X_3_ = Chitosan: TPP (*w*/*w*) (1:0.46)) on the response variable (Encapsulation Efficiency) and (**f**) 2D contour graph.

Accordingly, to equation 1, with the lowest pH solution (pH = 4.0) of X_1_, X_2_, and X_3_, a consistent increase in formulation encapsulation efficiency was observed, and this is certainly due to the formulation parameters [42,45]. The equation effect was emphasized and represented by a 3D-response surface and 2D contour graph plot as portrayed in Figure 1. The lowest in chitosan-CS/sodium Tripolyphosphate—TTP ratio CS/TPP (*w*/*w*), from (Figure 1a,c), increases in encapsulation efficiency. The combined effect of both pH (X_1_) and CS: TPP (X_3_) was positive for the response variable (Figure 1c,e), and the effect of pH (X_1_) and CS: ECO (X_2_) was negative on the response variable (Figure 1a).

**Figure 2:**
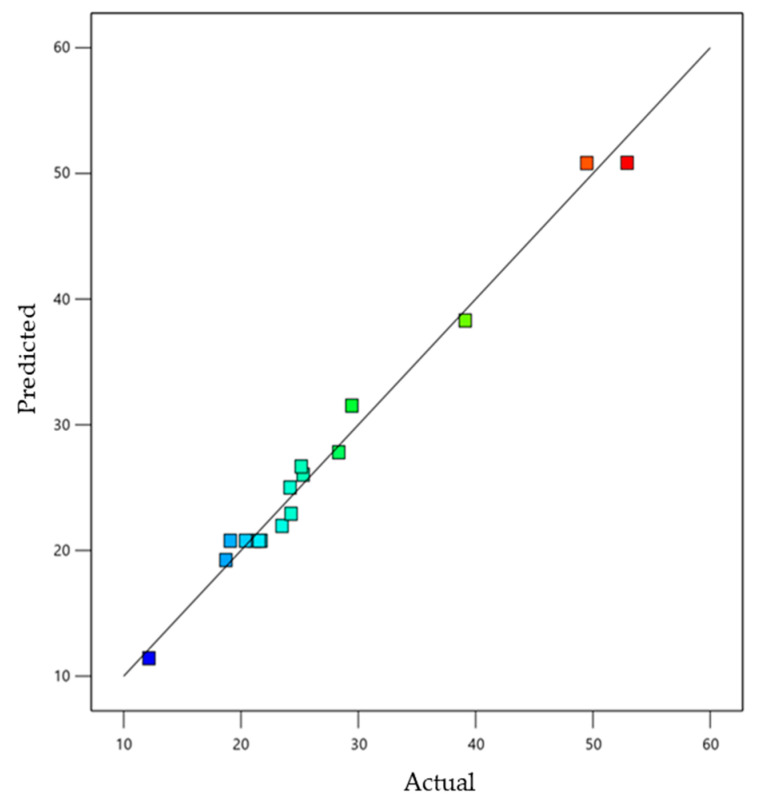
Linear correlation plot between actual and predicted values for Encapsulation Efficiency response (Y_1_).

As shown in Figure 2, a linear correlation was found between the predicted vs. the actual responses, where the predicted R^2^ for response Y_1_ was (0.82), which was in acceptable concordance with the adjusted R^2^ (0.9698), as can be observed in Table 2. R^2^ value (0.9868) also indicates that it may recommend the quadratic model, adding to that the adequate precision (27.49), which is a desirable value elucidating an adequate signal and which shows the model’s robust credibility. In addition, Figure 2 shows the residual values that were distributed between the two sides of the line, signifying that the actual data and the predicted values were in a credible correlation with each other.

### 2.3. Effect of Independent Variables on Loading Capacity (Y_2_)

The loading capacity corresponds to the final CEO concentration in 100% of the constituent’s amount utilized to synthesize the nanoparticles. As shown in Table 1, the loading capacity of the encapsulated chincho essential oil in the chitosan nanoparticles organogel system was determined, and the values ranged from 3.86% to 12.97%. These results were found to be in concordance with those reported by Keawchaoon and Yoksan [46], who informed on the loading capacity of carvacrol encapsulation in the value ranges 3–21% and 14–31%. Similarly, Shetta et al. [29] reported loading capacity values of encapsulated oils (peppermint and green tea essential oils) ranged from 8.15 to 22.2% and 2.2 to 23.1%. The features of the in loading capacity contents of CEO from the chitosan nanoparticles organogel system formulations were evaluated, and the result is shown in Figure 2. In encapsulated CEO, the results indicated that loading capacity increased as a function of the initial EO content, reaching their maximum level at 1:1 *w*/*w* with the EOs. The achieved values showed a good loading capacity, which shows that the CEO affinity with the wall material (chitosan), and the methodology used, was efficient to obtain CEO-CSNPs [47]. Figure 2e shows a considerable increase in the in loading capacity of CEO from the chitosan nanoparticles organogel system formulations, which was observed while increasing the concentration of independent factors X_2_, and X_3_. The Equation (2) interprets the previously stated influence of the independent variables X_1_, X_2_, and X_3_ on the loading capacity response Y_2_. 

Loading Capacity (Y_2_):

5.58 − 1.56 X_1_ + 1.03 X_2_ − 1.91 X_3_ − 0.9250 X_1_X_2_ + 1.32 X_1_X_3_ + 0.6575X_2_X_3_ −1.21 X_1_^2^ + 1.31 X_2_^2^ + 3.45 X_3_^2^ (2)

**Figure 3:**
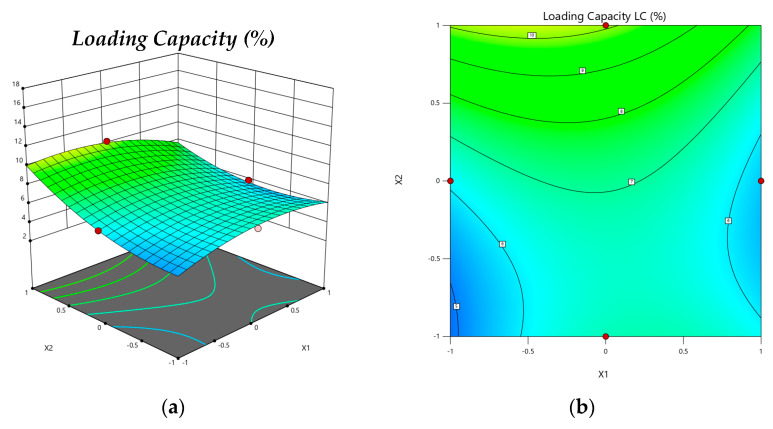

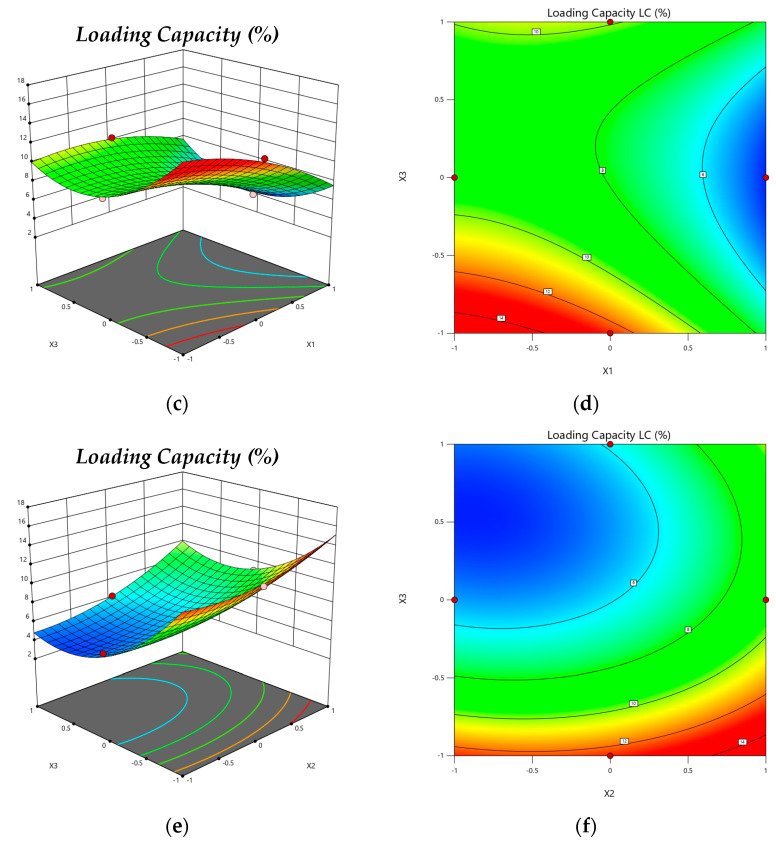
Model graphs: Loading Capacity. (**a**) Three-dimensional response surface plots showing the effect of variables (X_1_ = pH (4.4) and X_2_ = Chitosan: Chincho essential Oil (*w*/*v*) (1:0.7)) on response (Loading Capacity), (**b**) 2D contour graph, (**c**) 3D response surface plots showing the effect of variables (X_1_ = pH (4.4) and X_3_ = Chitosan: TPP (*w*/*w*) (1:0.46)) on the response (Loading Capacity), (**d**) 2D contour graph, (**e**) 3D response surface plots showing the effect of variables (X_2_ = Chitosan: TPP (*w*/*w*) (1: 0.7) and X_3_ = Chitosan: TPP (*w*/*w*) (1:0.46)) on response (Loading Capacity) and (**f**) 2D contour graph.

**Figure 4:**
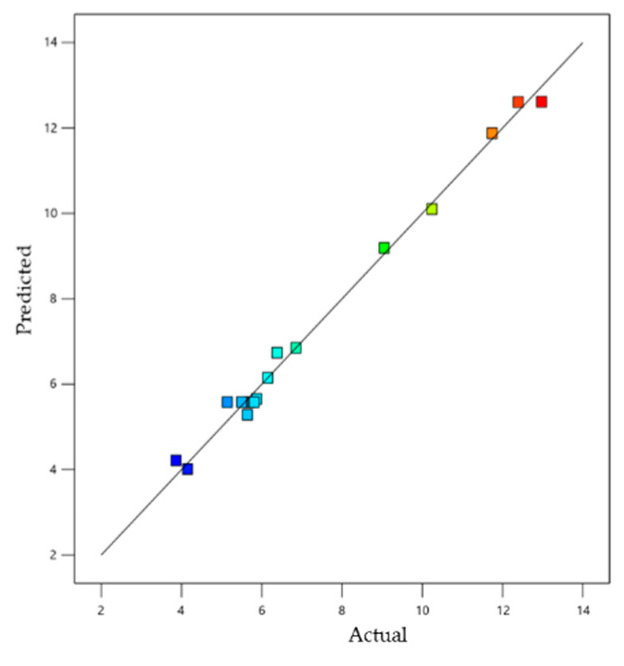
Linear correlation plot between actual and predicted values for loading capacity response (Y_2_).

As shown in Figure 3, this effect of the Equation (2) was represented by 3D-response surface and 2D contour graph plot. Figure 4, there was a linear relationship between the predicted and the actual responses, since the predicted R^2^ (0.9159) was found to be in a sensible harmony with the adjusted one (0.9834), because the data are similar, as demonstrated in Table 2. In addition, the value of R^2^ (0.9927) indicates that the system could support the model and the adequate precision value (30.12), which is a recommended value for the design space.

### 2.4. Optimization of the Encapsulation Process

Optimized CEO-CSNPs were produced using the identical methodology followed for all 17 trial formulations. The optimized formulation was prepared on the basis of values given by the Design Expert (Design Expert^®^ version 13 software, State Ease Incorporation, Minneapolis, MN, USA) after the analysis of 17 trial formulations for independent variables. The optimization process depends on pointing the responses (Y_1_, Y_2_) toward certain criteria that are expected to alter the optimized formula. The selected criteria in our study were to maximize the encapsulation efficiency and maximize loading capacity. The actual values calculated after optimization for the pH (X_1_), Chitosan: Chincho essential Oil (*w*/*v*) ratio (X_2_), and Chitosan: TPP (*w*/*w*)) ratio (X_3_) were 4.4, 1:0.7 (*w*/*v*), and 1:0.46 (*w*/*w*), respectively. The predicted values of the responses for optimization based on higher desirability were 50.85 ± 1.86 for Y_1_(%), and 11.87 ± 0.37 for Y_2_ (%). The experimental results are presented in Table 3. When the optimized formulation of CEO-CSNPs was prepared using actual values of independent variables, the responses’ actual values were 52.64 ± 2.44 for Y_1_ (%), and 11.56 ± 0.55 for Y_2_ (%). The responses’ actual values were close to the predicted values and were conspicuously close to each other. The optimized CEO-CSNPs were processed for characterization (zeta potential, PDI, hydrodynamic particle size, particle morphology), and were also characterized Scanning Electron Microscopy (SEM), in vitro release study, and antimicrobial activity.

### 2.5. Characterization of Optimized Formulations

#### 2.5.1. Particle Size, Zeta Potential, and Polydispersity Index PDI

The hydrodynamic nanoparticle size of CSNPs and CEO-CSNPs was examined, and the obtained values were the average of three independent measurements. The mean hydrodynamic particle sizes, Zeta Potential, and PDI of CSNPs were 284.85 ± 1.04, 21.49 ± 1.64 and 0.359 ± 0.01 nm respectively (Table 4).

**Figure 5:**
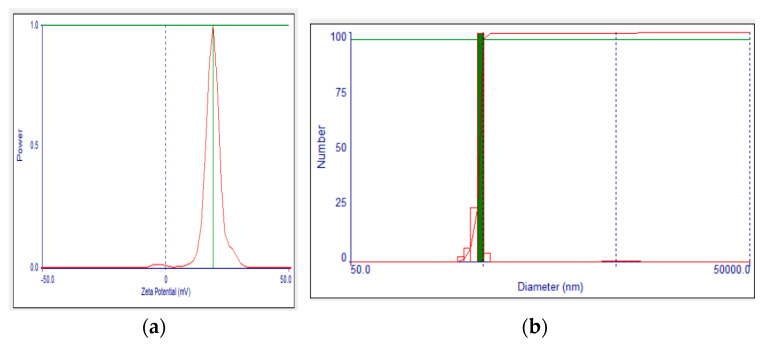
Average particle size distribution (**a**) and zeta potential graphs (**b**) of optimized CEO-CSNPs.

The hydrodynamic nanoparticle size of CSNPs and CEO-CSNPs was examined, and the obtained values were the average of three independent measurements. The mean hydrodynamic particle sizes, Zeta Potential, and PDI of CSNPs were 284.85 ± 1.04, 21.49 ± 1.64 and 0.359 ± 0.01 nm, respectively (Table 4). The particle size and Zeta potential of the optimized CEO-CSNPs were 458.5 ± 0.06 nm and 23.30 ± 2.15 mV, respectively (Figure 5). The incorporation of CEO significantly influences the size of nanoparticles. On the other side, the values obtained reveal no considerable variation in the zeta potentials of the formulations, which could be linked with the uncharged chemical nature of chincho essential oil [48]. The PDI values of CEO-CSNPs were 0.42 higher (0.2), which suggest a measure of the non-uniformity that exists in the particle size distribution [28] (Table 4).

#### 2.5.2. Surface Morphology

**Figure 6:**
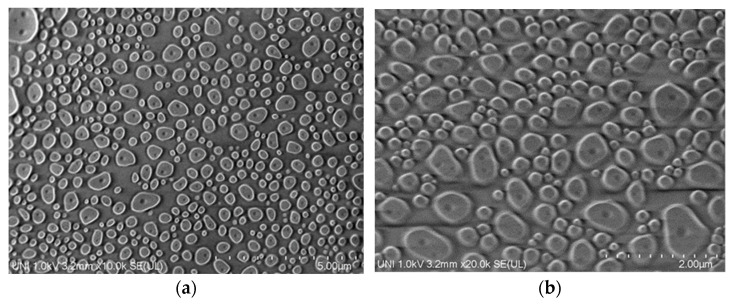

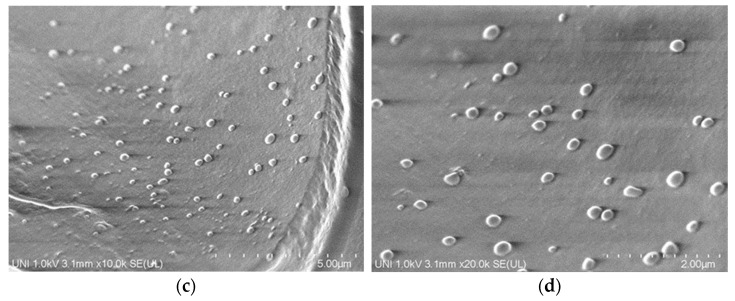
Scanning electron microscope (SEM) micrographs of CSNP at (**a**) 1000× magnification and (**b**) 20,000× magnification, and the optimized CEO-CSNPs at (**c**) 1000× magnification and (**d**) 20,000× magnification.

A scanning electron microscope (SEM) was used for the morphological analysis of the CS (Figure 6a,b) and CEO-CSNPs (Figure 6c,d) prepared by the ionic gelation method. For the nanoencapsulation of CS particles, no agglomeration of the nanoparticles organogel system was observed, as can be seen in Figure 6a,b. Surface morphology results were similar to those reported by Pinho Neves et al. [45]. In addition, it can be seen from the transmission electron micrograph results the nanoparticles CEO (Figure 6c,d) show a rounded and uniform shape, similar to that reported by [49] for essential oil *Origanum vulgare* and [50] for some natural extracts encapsulated in chitosan by the ionic gelation method using tripolyphosphate. The results showed that the nanoparticles organogel system prepared with this optimized formulation did not show aggregation between them, which was related to the charge on the surface of the encapsulates and the determined Z potential (23.30 ± 2.15 mV) (Table 4), which predicts good stability [29]. Morphological analysis by SEM showed an average CEO-CSNPs diameter of about 458 nm (Table 4), which validates the above results obtained with dynamic light scattering. A view at 10,000× and 22,000× (Figure 6c,d) allowed the observation of a dispersion inside the nanoparticle, which probably could mean the distribution of CEO formed in the matrix. The views were similar to what was observed by [51] in lyophilized chitosan hydrogel. Scanning electron microscopy is a good technique for determining the particles’ morphological characteristics in these tridimensional structures [52], such as organogels and nanoparticles. This study was similar than those carried out by Li et al. [53] and Glowka et al. [54] in which micro-organogels and nanoparticles, respectively, were sphere-like. 

#### 2.5.3. Release of Chincho Essential Oil at In Vitro Conditions

**Figure 7:**
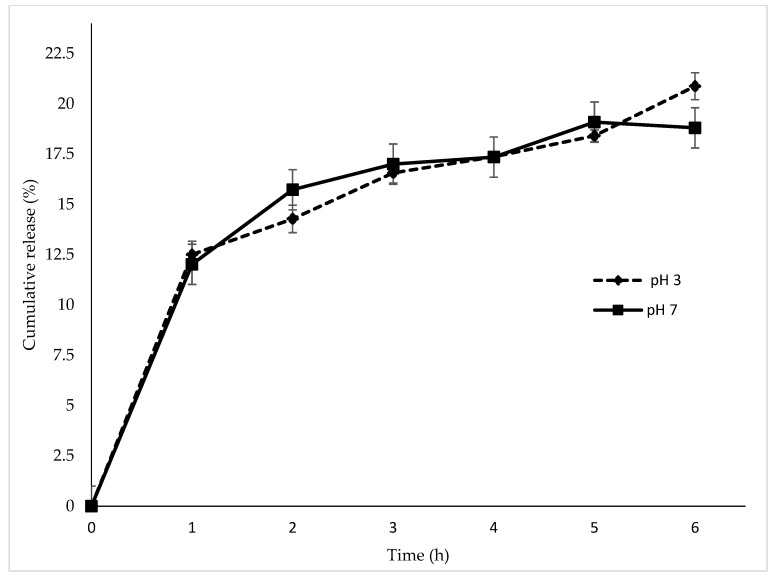
Chincho essential oil released (%) from chitosan nanoparticles organogel system obtained from optimization process in acetate and phosphate buffers (pH 3 and pH 7).

The in vitro release study of CEO from CEO-CSNPs was carried out for 6 h in different pH buffers (3 and 7). As shown in (Figure 7) the cumulative release rate of the CEO-CSNPs in acetate buffer (pH 3) from 0 to 6 h was 22.54 ± 0.66%, while that in phosphate buffer (pH 7) was only 18.80 ± 0.77%, demonstrating the slow-release characteristic of the CEO-CSNPs. The release of the active ingredients from the CEO is is partly due to the low mechanical strength of chitosan/TPP nanoparticles organogel system, and the release can be minimized by increasing the mechanical strength of the particle [55]. Controlled release is related to the increase in polymer concentration; the food might have better protection leading to a delay in the bioactive compound release [56]. Gallo et al. [57] elaborated oregano essential oil, loaded coated alginate beads nanoparticles in the oil release kinetics in a liquid medium, simulating a meat marinating solution, stating that the nanoparticles could be utilized as a natural antibacterial agent In addition, it is essential to know the effect of these processes in the application of food, due to these factors affecting its release profile and reducing or extending its effect. Organogels are semi-solids and have various unique characteristics, such as surface lubricity and anti-drying capacity, arousing particular interests in diverse practical applications. They are used as delivery systems, but are relatively new in food science. They have high stability and high encapsulation efficiency [58]. To obtain them, there are methods ranging from the basic composition to gelation mechanism, and fabrication strategies, such as the ionic gelation method [59]. In addition, Corredor et al. [60] determined that sesame oil organogels have a controlled release and greater stability of their active molecules.

#### 2.5.4. Antimicrobial Activity

The minimum inhibitory concentration (MIC) values for chitosan encapsulated CEO, measured against *S. aureus*, *E. coli* and *S. infantis*, are shown in Table 5. The antimicrobial activity for CEO-CSNPs reported MIC values in the range of 5.29–10.57 µg/mL, and presented better activity against Gram-positive bacteria. The chitosan nanoparticles organogel system, as well, showed better antibacterial action against Gram-positive bacteria than Gram-negative bacteria, according to the findings in the range of 21.14–42.29 µg/mL. This has been associated with its ability to bind non-covalently with teichoic acids incorporated in the peptidoglycan layer of the bacteria [61]. As far as our literature survey could ascertain, *T. elliptica* Sm. essential oil MIC 5 µL/mL was reported against microorganisms *Staphylococcus aureus* [18], with values similar to those reported in this study; the antimicrobial activity of CEO-CSNPs can be associated with their particle size [62]. The antimicrobial activity of chincho essential oil has been attributed mainly to the presence of phenolic compounds (β-myrcene, cis-lanalool oxide and 2-tujene) [20]. The antimicrobial activity obtained from CEO-CSNPs suggest that they act as a physical delivery system. Antimicrobial compounds, among other compounds during the elaboration process, were kept. These results agreed with those obtained by Chen et al. [58]. 

## 3. Conclusions

The 3-factor, 3-level Box–Benhken design used provided fitting polynomial equations for the evaluated responses (EE and LC), and was consequently used with success to optimize the chincho essential oil nanoparticle formulation. The optimized NPs were reproducible with a particle size of 458.5 ± 0.06 and with a ZP of 23.30 ± 2.15 mV and polydispersity index of 0.418 ± 0.02. Morphological studies of optimized CEO-CSNPs revealed the rounded and uniform shape of the particles. In vitro release studies of CEO-CSNPs formulation were around 18.80 ± 0.77% and 22.54 ± 0.66% over 6 h. CEO-CSNPs act as physical delivery system for CEO antimicrobials. The minimum inhibitory concentrations (MIC) of CEO-CSNPs against three bacterial strains, *Staphylococcus aureus* (5.29 µg/mL), *Salmonella infantis* (10.57 µg/mL), and *Escherichia coli* (10.57 µg/mL), were determined. The size of the NPs in conjunction with the antibacterial properties of the chincho essential oil suggest these particles might be promising as a food additive. However, the obtained nanoparticles organogel system require subsequent shelf-life study and a food application in a different matrix. Thus, it really could be evaluated for its potential use in the food industries.

## 4. Materials and Methods

### 4.1. Materials

Chincho essential oil (CEO), the essential oil of chincho (CEO), was extracted from leaves by hydro-distillation using a Clevenger-type apparatus for 3 h. The leaves were collected in Junin Region, Peru (3263 m.a.s.l.). Chitosan (CS) (high molecular weight, deacetylated chitin, PolyCD-glucosamine), Tween 80, Sodium Hydroxide and TPP were purchased from Sigma-Aldrich (Burlington, MA, USA). Sodium Acetate Anhydrous ACS (Fermont, Canada), Potassium Phosphate Monobasic (Panreac, Barcelona, Spain), and di-Sodium Hydrogen Phosphate anhydrous purest anhydrous (Panreac, Barcelona, Spain) were also used. The bacterial strains *Staphylococcus aureus* ATCC 25923TM, *Escherichia coli* ATCC 25922TM, and *Salmonella infantis* were obtained from Calidad Total and the Food Microbiology Laboratory (Universidad Nacional Agraria La Molina, Lima, Peru). Methanol and acetic acid (glacial) were acquired from Merck (Darmstadt, Germany).

### 4.2. Experimental Design Using Box–Benhken (BBD)

In order to obtain chincho essential oil nanoparticles, a matrix of 17 formulations was constructed and fabricated by RSM. Design Expert (Design Expert^®^ version 13 software, State Ease Incorporation, Minneapolis, MN, USA) was used for the optimization of the formulation using a Box–Behnken design (BBD) via constructing three factors and three levels as shown in Table 6. Thus, BBD was applied using chitosan solution (CS) pH (4.0, 4.4, 4.8); the mass ratio of CS/CEO (1:0.7, 1:0.85, 1:1.0); and the mass ratio of CS/CS-TPP (1:0.46, 1:0.58, 1:0.7) as independent variables. The formulation-dependent variables, encapsulation efficiency (EE) and loading capacity (LC) of the CEO-CSNPs, were evaluated. In this study, the factors representing the independent variables selected were X_1_, X_2_, and X_3_, related to pH of the CS solution, the CS/CEO mass ratio and, the CS/TPP mass ratio, respectively. The influence of the above factors was assessed on the responses Y_1_ (encapsulation efficiency) and Y_2_ (loading capacity) of the elaboration chincho essential oil nanoparticles. Statistical analysis of the values obtained could be determined via the analysis of variance (ANOVA) test. The interaction of independent variables and responses was determined using the following quadratic mathematical model, where Y represents the detected response (Equation (3)):

Y = b_0_ + b_1_X_1_ + b_2_X_2_ + b_3_X_3_ + b_1,2_X_1_X_2_ + b_1,3_X_1_X_3_ + b_2,3_X_2_X_3_+ b_1,1_X_1_^2^ + b_2,2_X_2_^2^ + b_3,3_X_3_^2^ (3)

The optimized formulation of chincho essential oil nanoparticles were selected on the basis of factor desirability over responses.

### 4.3. Preparation of CEO-CSNPs by Ionic Gelation

Ionic gelation methodology, based in the recommendations reported by Zhang et al. [37], was used to elaborate the chitosan nanoparticles loaded with chincho essential oil (CEO-CNPs). A two-step process was applied. The first phase was oil-in-water (o/w) emulsification and the second one was ionic gelation. Chitosan (CS) solution (3.2 mg/mL) was prepared by dissolving in acetic acid solution (1% (*v*/*v*)) using a magnetic stirrer at 60 °C for 60 min. Then, the CS solution was filtered through a 1-μm pore size filter to remove any undissolved CS. Once dissolved, all chitosan solutions were divided into three equal volumes and the pH of the solutions was adjusted to pH: 4.0; 4.4, and 4.8. Tween-80 (1.6 mg/mL) was used as a surfactant agent, and the solutions was stirred for 30 min at 18 ºC until a homogeneous mixture was obtained. Three different volumes of chincho essential oil (126.65; 153.8; 180.95 μL) were dissolved in ethanol (4 mL) to obtain CS to CEO weight ratios of 1:0.7; 1:0.85 and 1:1 (mg/mL), respectively, forming the oily phase of the emulsion. After cooling, the oil phase was progressively dropped into the aqueous phase under vigorous stirring, and agitation was continued for 20 min. After that, three different volumes of 30 mL of the TPP solution were used to obtain CS to TPP weight ratio of 1:0.46; 1:0.58 and 1:1 (mg/mL) and were added to the solution, which was mixed for 25 min using a magnetic stirrer. To guarantee the complete gelation, mixing was continued for 45 min after the complete addition of the tripolyphosphate. The cross-linked NPs were collected by centrifugation for 30 min at 6000 rpm at 20 °C and stored at 4 °C until use.

#### Determination of Encapsulation Efficiency (EE%) and Loading Capacity (LC%)

To determine the encapsulation efficiency (EE%), encapsulated CEO was determined by UV–Vis spectrophotometry. Predetermined amounts of CEO-CSNPs were dispersed into 3 mL methanol and centrifuged at 6000 rpm for 15 min at 18 °C from the aqueous medium containing non-associated oil as reported Natrajan et al. [24]. The CEO present in the supernatant was determined using spectrophotometer UV7 (Mettler Toledo, Barcelona, Spain) at a wavelength of 230 nm. The CEO concentration was determined by a proper calibration curve of pure CEO and CEO in methanol with R^2^ of 0.9909 for the EO. Chitosan nanoparticles were treated in the same way, and they were used as a blank. Triplicate samples for each batch were recorded. Encapsulation efficiency (EE%) and loading capacity (LC%) were estimated from Equations (4) and (5) respectively.

(4) $$\mathrm{Encapsulation}\;\mathrm{Efficiency}\;\left(\mathrm{EE}\%\right)=\frac{\mathrm{Total}\;\mathrm{amount}\;\mathrm{of}\;\mathrm{loaded}\;\mathrm{CEO}}{\mathrm{Initial}\;\mathrm{amount}\;\mathrm{of}\;\mathrm{CEO}}\times 100$$

(5) $$\mathrm{Loading}\;\mathrm{Capacity}\;\left(\mathrm{LC}\%\right)=\frac{\mathrm{Total}\;\mathrm{amount}\;\mathrm{of}\;\mathrm{loaded}\;\mathrm{CEO}}{\mathrm{Weight}\;\mathrm{of}\;\mathrm{nanoparticles}\;\mathrm{constituents}}\times 100$$

### 4.4. Characterization of Chincho (Tagetes ellitptica Sm.) Essential Oil-Loaded CS-NPs

#### Polydispersity, Particle Size, and Zeta Potential

Dynamic light scattering (DLS) on the 90 Plus/BI-MAS (Brookhaven Instruments Corporation, Holtsville, NY, USA) was utilized to determine the mean particle size (Z-average), polydispersity index, and zeta potential of CEO-CSNPs in the hydrated state. To produce an average, light scattering was measured in triplicate at a 90 degree angle and at 25 °C. On the 90 Plus/BI-MAS, the zeta potential was assessed by means of an electrophoretic light scattering approach. The CEO-CSNPs samples were diluted with 1 mM potassium chloride before being located in the electrophoretic cell. The mean electrophoretic mobility data were used to determine the zeta potential values [40], with slight modifications. All measurements were performed in triplicate, and results were expressed as mean ±standard deviation (SD).

### 4.5. Particles Morphology

The optimized CEO-CSNPs morphology was determined using a scanning electron microscope (Hitachi High-Tech, SU8230, Hitachi High-Technologies Corporation, Tokyo, Japan). Before the scanning electron microscope analysis, the optimized CEO-CSNPs were lyophilized using a freeze-dryer Lyovapor L-200 (Buchi iberica, Barcelona, Spain). Lyophilized nanoparticles were assembled on aluminum stubs held by coal adhesive tape. The scanning electron microscope was used to visualize the morphology of the CEO-CSNPs under high vacuum at 10 kV accelerated voltage [63].

### 4.6. In Vitro Release Study

The release properties of CEO from chitosan nanoparticles were determined using two buffer solutions at pH3 (acetate buffer) and pH 7 (phosphate buffer). Sample dispersions (500 μL) were centrifuged at 2350× *g* for 8 min at 18 °C. Water was removed and the buffer solution (3 mL) was added into a centrifuge tube containing nanoparticles. The mixture was agitated (1 min in a vortex) and incubated at 22 °C during six hours. At different incubation time intervals (1 h), samples were centrifuged at 2350× *g* for 8 min at 18 °C, and a specific volume of supernatant was then taken for analysis and replaced with an equal volume of fresh buffer. The amount of CEO released in the supernatant was analyzed by a spectrophotometer over wavelengths from 230 nm, following the recommendations of Keawchaoon and Yoksan [46].

### 4.7. Antibacterial Study

The broth microdilution method was used to determine the minimum inhibitory concentration (MIC) following the methodology described by Alves-Silva et al. [64]. Three bacteria strains, *Staphylococcus aureus* ATCC 25923TM, *Escherichia coli* ATCC 25922TM, and *Salmonella infantis*, were utilized as test microorganisms. All bacteria strains were cultured for 24 h at 37 °C in Mueller Hinton broth (MHB). The concentration of culture suspensions was adjusted to 10^6^ CFU/mL by comparison with McFarland turbidity. Serial twofold dilutions were made, and bacterial suspensions (100 μL) were then inoculated in the sample series. After incubation at 37 ° C for 24 h, MIC was determined as the concentration of the sample in the tube without turbidity and containing the lowest sample concentration. Chitosan nanoparticles were used as a positive control, whilst the blank control only had MH broth and bacterial inoculum. Tests were performed in triplicate for each sample.

### 4.8. Statistical Analysis

Statistical analysis was performed, and the data are expressed as mean ± SD from three separate observations. The data from the Box–Behnken experimental design were analyzed by least square multiple regression methodology to fit the polynomial models in CEO-CSNPs optimization. Data analysis and response surfaces were conducted using the software Design Expert (Design Expert^®^ version 13 software, State Ease Incorporation, Minneapolis, MN, USA).

## Figures and Tables

**Table 1:** Box–Benhken matrix and responses for the different formulations under study.

Run	Independent Variables			Dependent Response	
	X_1_	X_2_	X_3_	Y_1_	Y_2_
1	0	0	0	21.55 ± 1.31	5.81 ± 0.37
2	1	0	1	24.27 ± 0.60	5.87 ± 0.29
3	0	0	0	19.09 ± 0.85	5.14 ± 0.23
4	−1	0	1	23.50 ± 1.39	6.15 ± 0.20
5	1	−1	0	18.71 ± 0.53	4.15 ± 0.10
6	0	1	1	29.46 ± 0.63	10.24 ± 0.44
7	0	1	−1	39.12 ± 0.96	12.97 ± 1.19
8	1	1	0	12.15 ± 0.44	3.86 ± 0.13
9	−1	1	0	28.34 ± 3.10	9.05 ± 1.01
10	0	0	0	21.71 ± 0.44	5.77 ± 0.22
11	0	0	0	20.38 ± 0.69	5.50 ± 0.20
12	0	0	0	21.17 ± 1.02	5.68 ± 0.30
13	0	−1	1	24.19 ± 0.41	6.38 ± 0.73
14	−1	0	−1	49.48 ± 0.80	12.39 ± 1.69
15	−1	−1	0	25.31 ± 4.38	5.64 ± 0.69
16	1	0	−1	25.14 ± 1.49	6.85 ± 0.43
17	0	−1	−1	52.92 ± 0.65	11.74 ± 0.87

X_1_ = chitosan-CS solution pH, X_2_ = Chitosan-CS/chincho essential Oil-CEO (*w*/*v*), X_3_ = Chitosan/sodium Tripolyphosphate-TPP (*w*/*w*), Y_1_ = Encapsulation Efficiency EE (%), Y_2_ = Loading Capacity LC (%).

**Table 2:** Adequacy of the model and model summary statistics for encapsulation efficiency and loading capacity.

Source	Y_1_		Y_2_	
	F-Value	*p*-Value Prob > F	F-Value	*p*-Value Prob > F
Model	58.10	<0.0001 *	106.09	<0.0001 *
X_1_	76.84	<0.0001 *	140.78	<0.0001 *
X_2_	5.20	0.0566	60.73	0.0001 *
X_3_	152.17	<0.0001 *	211.18	<0.0001 *
X_1_X_2_	6.58	0.0373 *	24.67	0.0016 *
X_1_X_3_	45.09	0.0003 *	49.86	0.0002 *
X_2_X_3_	26.00	0.0014 *	12.46	0.0096 *
X_1_^2^	9.03	0.0198 *	44.53	0.0003 *
X_2_^2^	11.47	0.0116 *	51.78	0.0002 *
X_3_^2^	189.87	<0.0001 *	360.44	<0.0001 *
Lack of Fit	5.72	0.0626 not significant	3.00	0.1583 not significant
R^2^ analysis				
R^2^	0.9868		0.9927	
Adjusted R^2^	0.9698		0.9834	
Predicted R^2^	0.8247		0.9159	
Adequate Precision	27.4914		30.1171	
Model				
Remark	Quadratic		Quadratic	

X_1_ = chitosan-CS solution pH, X_2_ = Chitosan-CS/chincho essential Oil-CEO mass ratio CS/CEO (*w*/*v*), X_3_ = chitosan-CS/sodium Tripolyphosphate—TTP ratio CS/TPP (*w*/*w*), Y_1_ = Encapsulation Efficiency EE (%), Y_2_ = Loading Capacity LC (%). * There were significant differences.

**Table 3:** The response of predicted and experimental values of the optimizated conditions.

Response	Predicted Values	Experimental Values
Y_1_ (%)	50.85 ± 1.86	52.64 ± 2.44
Y_2_ (%)	11.87 ± 0.37	11.56 ± 0.55

Y_1_ = Encapsulation Efficiency EE (%), Y_2_ = Loading Capacity LC (%).

**Table 4:** Characterization of the optimized CEO-loaded CSNPs in terms of encapsulation efficiency, loading capacity, mean hydrodynamic particle size, polydispersity index (PDI), and zeta potential.

Formulation	%EE	%LC	Particle Size (nm)	Zeta Potential (mV)	PDI
CSNP	--	--	284.85 ± 1.04	21.49 ± 1.64	0.359 ± 0.01
CEO-CSNPs	52.64 ± 2.44	11.56 ± 0.55	458.50 ± 0.06	23.30 ± 2.15	0.418 ± 0.02

Data expressed as mean ± SD (n = 3); CSNP: chitosan nanoparticles organogel system; CEO-CSNPs: chincho essential oil chitosan nanoparticles organogel system.

**Table 5:** Antimicrobial activity of CSNPs and CEO-CSNPs, expressed as minimum inhibitory concentration (MIC) against *S. aureus*, *E. coli*, and S. *infantis*.

	Antimicrobial Activity MIC (µg/mL)		
	*S. aureus* ATCC 25923	*E. coli* ATCC 25922	*S.* *infantis*
CSNPs	21.14	42.29	42.29
CEO-CSNPs	5.29	10.57	10.57

CSNP: chitosan nanoparticles organogel system; CEO-CSNPs: chincho essential oil chitosan nanoparticles organogel system.

**Table 6:** Independent and dependent variables of the 3-level, 3-factor Box–Behnken design.

**Independent Variable**	**Coded Levels**		
	**Low Level**	**Medium Level**	**High Level**
	−1	0	1
X_1_: pH	4.0	4.4	4.8
X_2_: Chitosan: Chincho essential Oil-CS:CEO (*w*/*v*)	1:0.7	1:0.85	1:1
X_3_: Chitosan: TPP—CS:TPP (*w*/*w*)	1:0.46	1:0.58	1:0.7
**Dependent Variables**	**Criteria**		
Y_1_: Encapsulation Efficiency EE (%)	Maximum		
Y_2_: Loading Capacity (%)	Maximum		

## Data Availability

The data presented in this study are available on request from the corresponding author.
